# Enhancing Cognitive Abilities with Comprehensive Training: A Large, Online, Randomized, Active-Controlled Trial

**DOI:** 10.1371/journal.pone.0134467

**Published:** 2015-09-02

**Authors:** Joseph L. Hardy, Rolf A. Nelson, Moriah E. Thomason, Daniel A. Sternberg, Kiefer Katovich, Faraz Farzin, Michael Scanlon

**Affiliations:** 1 Department of Research and Development, Lumos Labs, San Francisco, California, United States of America; 2 Department of Psychology, Wheaton College, Norton, Massachusetts, United States of America; 3 Merrill Palmer Skillman Institute for Child and Family Development, Wayne State University, Detroit, Michigan, United States of America; 4 Department of Pediatrics, Wayne State University School of Medicine, Detroit, Michigan, United States of America; University of Regensburg, GERMANY

## Abstract

**Background:**

A variety of studies have demonstrated gains in cognitive ability following cognitive training interventions. However, other studies have not shown such gains, and questions remain regarding the efficacy of specific cognitive training interventions. Cognitive training research often involves programs made up of just one or a few exercises, targeting limited and specific cognitive endpoints. In addition, cognitive training studies typically involve small samples that may be insufficient for reliable measurement of change. Other studies have utilized training periods that were too short to generate reliable gains in cognitive performance.

**Methods:**

The present study evaluated an online cognitive training program comprised of 49 exercises targeting a variety of cognitive capacities. The cognitive training program was compared to an active control condition in which participants completed crossword puzzles. All participants were recruited, trained, and tested online (N = 4,715 fully evaluable participants). Participants in both groups were instructed to complete one approximately 15-minute session at least 5 days per week for 10 weeks.

**Results:**

Participants randomly assigned to the treatment group improved significantly more on the primary outcome measure, an aggregate measure of neuropsychological performance, than did the active control group (Cohen’s *d* effect size = 0.255; 95% confidence interval = [0.198, 0.312]). Treatment participants showed greater improvements than controls on speed of processing, short-term memory, working memory, problem solving, and fluid reasoning assessments. Participants in the treatment group also showed greater improvements on self-reported measures of cognitive functioning, particularly on those items related to concentration compared to the control group (Cohen’s *d* = 0.249; 95% confidence interval = [0.191, 0.306]).

**Conclusion:**

Taken together, these results indicate that a varied training program composed of a number of tasks targeted to different cognitive functions can show transfer to a wide range of untrained measures of cognitive performance.

**Trial Registration:**

ClinicalTrials.gov NCT-02367898

## Introduction

Recent evidence suggests that engaging in cognitively challenging activities can positively impact brain function, with studies demonstrating behavioral [1, 2], physiological [3, 4], and real-world functional [5, 6] gains. This notion is supported by growing empirical evidence that neuroplasticity–the tendency for the nervous system to adapt to environmental challenges presented to it–is a fundamental principle of brain organization [7–9].

New appreciation of the importance of neuroplasticity has led to the development of a variety of cognitive training programs–activities designed to elicit enhancements in cognitive abilities through intensive, targeted mental exercise. Several such programs have been used in research, with promising results for improving cognitive functioning following training reported in most cases [2, 4, 10–16]; however, other studies have failed to demonstrate such gains [17, 18]. Because cognitive abilities are critical for success at work [19], school [20–22], and activities of daily living [23], there is considerable interest in using large-scale approaches to rigorously investigate the efficacy of cognitive intervention strategies.

The present study enrolled participants via the Internet into either a cognitive training treatment condition or an active control condition. The treatment was the off-the-shelf version of Lumosity, an online cognitive training program, where participants trained on up to 49 tasks that were presented in game-like formats. Specific tasks within the program were designed to target particular cognitive abilities, such as speed of processing, working memory, divided attention, response inhibition, and fluid reasoning. Training tasks challenged users to operate close to their performance thresholds. A wide variety of tasks were used in training, reducing the opportunity for use of task-specific strategies. This variety increased the opportunity for “learning to learn,” which may enhance transfer to untrained tasks [24]. Previous studies using this program have demonstrated improvements in cognition in children with Turner’s Syndrome [25]; pediatric cancer survivors [26]; healthy middle-aged adults [27]; healthy older adults [28]; older adults with mild cognitive impairment [29]; and adult survivors of breast cancer [30].

The active control group in this study engaged in solving crossword puzzles. This activity was chosen because crossword puzzles constitute a challenging mental activity that is popularly believed to be beneficial for cognition [31]. Some health professionals specifically advocate the use of crossword puzzles for sharpening mental skills [32]. While there is relatively little experimental evidence supporting the efficacy of crossword puzzles, one observational study has linked regular engagement with crossword puzzles to a delay in the onset of memory decline in older adults [33].

The goal of this study was to measure the efficacy of a targeted, progressively challenging, comprehensive cognitive training program against a plausibly beneficial active control condition in a large, randomized trial. We hypothesized that this type of cognitive training would show greater transfer to a range of underlying cognitive abilities than the active control, as measured by a broad battery of neuropsychological assessments and participant-reported outcomes.

## Methods

### Ethics statement

Participants provided informed consent by clicking a dialogue box on a digital consent form prior to participation in the study. All study materials and procedures were approved by an independent institutional review board (Ethical and Independent Review Services; Corte Madera, CA). The IRB-approved study protocol is included as Supporting Information (S1 Protocol).

### Trial registration

The study was registered on ClinicalTrials.gov (NCT-02367898) upon the request of the journal staff. The investigators had not previously registered on the site, as the trial did not involve a clinical population. The authors confirm that all ongoing and related trials for this intervention are now registered.

### Participants

Participants were recruited from the Lumosity website (www.lumosity.com). Individuals who had created an account on the site, but who were not paying subscribers (i.e., free users) were eligible for recruitment. Invitations were sent via email to users who engaged with the program on at least three days in the first week after sign-up. All participants who completed the study were compensated with a 6-month membership to Lumosity.

A power analysis based on results from an open-label study of the treatment program, a portion of which has been presented previously [34], suggested that 5,000 participants (2,500 per group) would provide greater than 99% power to obtain significance on the primary outcome measure and greater than 70% to detect a dose-response interaction between groups, if one existed. Based on the ongoing study completion rate, recruitment ended when it was estimated that the number of participants enrolled in the study would be sufficient to obtain 5,000 fully evaluable participants.

**Fig 1:**
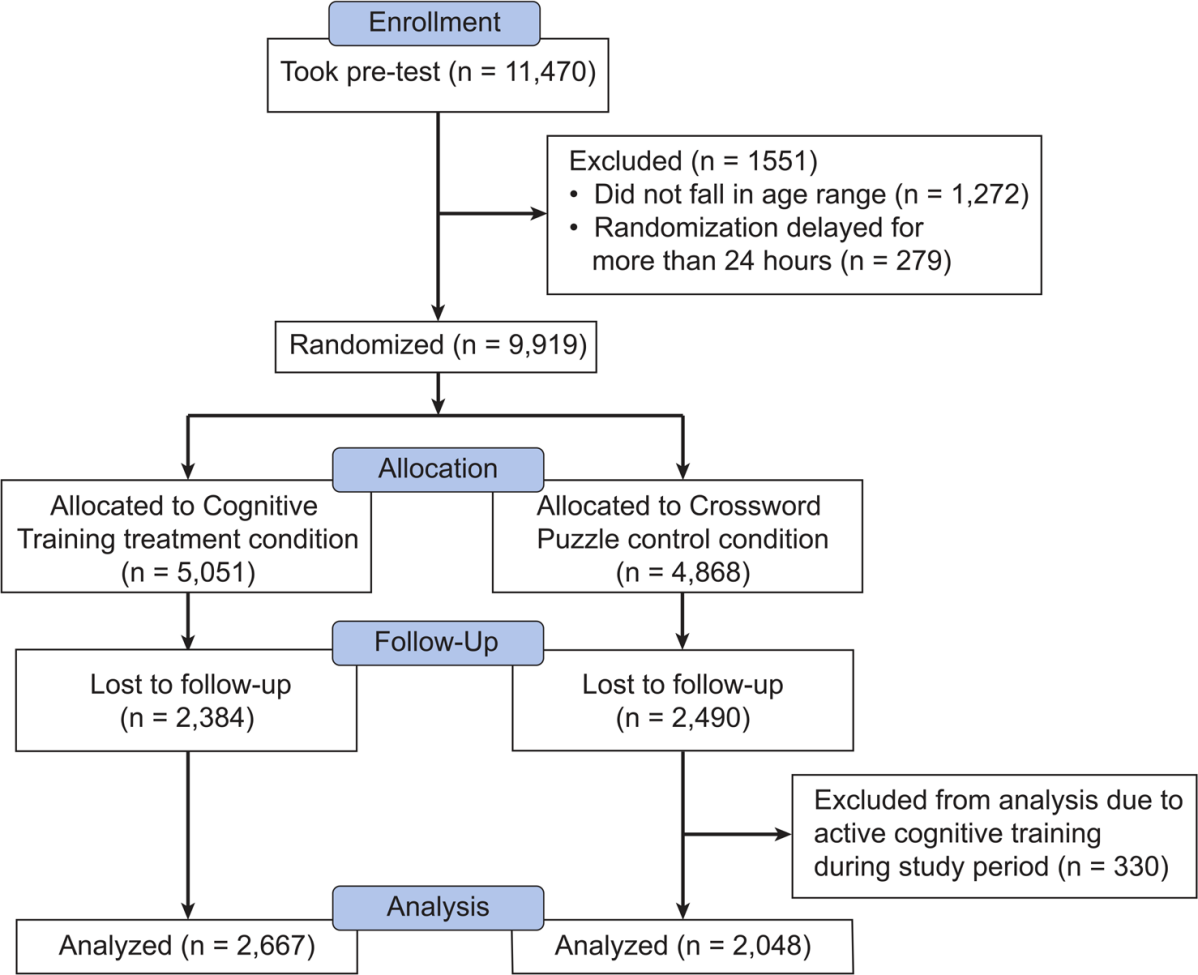
CONSORT flow chart of participants in the study.

In total, 11,470 individuals consented to take part in the study and completed a baseline (pre-test) assessment battery. The first participant was randomized on April 27, 2013, and the final participant completed the post-test on April 28, 2014. Participants were assigned a treatment condition using a random number generator with equal probabilities of assignment to cognitive training and crosswords control conditions. Random assignment occurred after the pre-test. Participants with age falling outside the target range of 18–80 were excluded prior to randomization (N = 1,272). An additional 279 participants were excluded because a computer error delayed their randomization into a treatment condition by more than 24 hours, allowing these participants to continue with the Lumosity program in the free user state. Of the remaining 9,919 participants randomized into a treatment condition, 5,045 (50.9%) completed the post-study assessment battery (post-test). The training platform was designed to direct each participant, upon logging in each day, to either cognitive training or crossword puzzles based on his or her group assignment. However, in some cases participants in the crossword control group were able to access cognitive training. As a result, 330 control participants were removed from the primary analysis because they accessed the cognitive training program during the study period (Fig 1). See Table 1 for demographic characteristics of the fully evaluable cohorts in both conditions. Age, gender, and educational attainment were evenly distributed across the groups.

**Table 1:** Demographic information for fully evaluable participants.

	Crosswords Control	Cognitive Training	p value
**Age**			
Mean age (sd)	39.0 (14.9)	39.4 (15.2)	0.58
% 18–34	50.9%	49.3%	
% 35–54	30.1%	30.6%	
% 55 and older	18.9%	20.1%	
**Gender** % Female (% unreported)	53.7% (2.1%)	54.4% (2.5%)	0.47
**Education**			0.40
% high school graduate or less	11.3%	11.3%	
% with some college	25.3%	23.2%	
% with a bachelor’s degree	31.4%	30.2%	
% with advanced degree (Masters, PhD, or Professional degree)	27.6%	28.9%	
% unreported	4.3%	6.3%	

P values are based on Kolmogorov-Smirnov test for age, chi-square for gender and education. Statistics for gender and education are based on participants who reported this information.

### Treatment and control groups

All participants were instructed to log into the website and do one session per day of their activity (cognitive training for the treatment group or crossword puzzles for the control group), 5 days a week for 10 weeks. Daily email participation reminders were sent to all participants during the study period.

#### Cognitive training treatment

The Lumosity cognitive training program was used as the treatment condition in this study. Treatment participants in this study received the same training experience that Lumosity subscribers received over the same period of time. Daily training sessions included five cognitive training tasks. On any given day, the five tasks for that particular session were chosen by an algorithm that attempted to optimize a balance of training activities such that tasks were presented in clusters across days without repeating individual tasks on a given day. One five-task session typically took approximately 15 minutes to complete. Outside of this session, participants could opt to do additional training with any of the 49 available tasks in an *a la carte* fashion.

The cognitive training tasks each target a particular core cognitive ability and are grouped into five categories by target domain: speed of processing, attention, memory, flexibility, and problem solving. Many of these tasks are described in detail elsewhere in the literature [25–27, 29, 30, 35–37], and a description of all tasks is included as Supporting Information (S1 Appendix).

#### Crossword puzzles control

Participants randomized into the active control group received a daily session timed at a minimum of 15 minutes. They were instructed to complete as many crossword puzzles as possible in the allotted time. If a participant completed a puzzle within the 15-minute time period, the crossword application would provide a new puzzle. At the end of the 15-minute period, participants were able to continue to work on the current puzzle for as long as they chose but were not given additional puzzles that day. The crossword puzzles were produced by professional crossword constructors and presented in a web-based crosswords platform. Constructors were asked to create crosswords that were of medium difficulty, approximately equivalent to a Thursday New York Times crossword puzzle (note: the New York Times puzzles increase in difficulty throughout the week, culminating with the most difficult puzzle on Saturday). Participants filled out the puzzles by typing the answers in the appropriate boxes. Feedback about correct and incorrect responses was given immediately following submission of a completed crossword. The puzzles were placed in a website frame that replicated the look and feel of the cognitive training website in order match as closely as possible the experience across the two conditions.

### Compliance

Compliance with the study protocol was assessed via two measures: (1) the number of unique days that treatment participants completed at least one training task or control participants started a crossword puzzle (“active days”), and (2) the estimated total time participants spent engaging with the respective condition. See the Supporting Information (S1 File) for additional details on how engagement time was estimated. As participants were instructed and reminded to complete daily sessions, the number of active days was used as the primary measure of a participant’s ongoing engagement and compliance with the study protocol. Secondary analyses based on total time are included in S1 File.

### Outcome measures

Outcomes were assessed using a battery of seven neuropsychological tests, as well as a participant-reported outcomes survey. The primary outcome measure used in this study was change in aggregate cognitive performance, as measured by the Grand Index (described further below) of the neuropsychological assessment battery, from before to after the 10-week study period. Secondary outcome measures included change in performance on each of the subtests in the neuropsychological battery and changes in responses to the survey. The assessments and survey were administered online in a pre-test one day prior to beginning the treatment or control condition. Participants were directed to take the post-test 70 days later, one day following the end of the treatment or control.

#### Neuropsychological assessment battery

Seven neuropsychological assessments were used in this study. (1) Forward and (2) Reverse Memory Span assessed visual short-term and working memory, respectively, and are based on the Corsi Blocks tasks [38]. These assessments required participants to recall a sequence of randomized spatial locations in either forward or reverse order. (3) Grammatical Reasoning was based on Baddeley’s Grammatical Reasoning Test [39], designed to assess cognitive flexibility and reasoning, and required participants to rapidly and accurately evaluate potentially confusing grammatical statements. (4) Progressive Matrices was based on established matrix reasoning assessments [40] and was designed to assess problem solving and fluid reasoning. (5) Go/No-Go was designed to assess response inhibition and processing speed, and required participants to respond as quickly as possible to a target stimulus while avoiding responding to distractors. (6) Arithmetic Reasoning was designed to assess numerical problem solving ability and required the participant to respond as quickly and accurately as possible to arithmetic problems written in words (e.g., “Four plus two =”) [41]. (7) Two-Target Search was created for the purposes of this study. This task was designed to measure divided visual attention and required participants to recall the locations of briefly presented target letters while ignoring distractors. See the Supporting Information (S2 Appendix) for more detailed information about the design of these assessments. Importantly, none of the tasks used in the outcome assessment battery were presented during training. Rather, outcome assessments were implemented as measures of transfer to underlying cognitive abilities.

#### Assessment scaling procedure

Our assessment scaling procedure follows standard rank-based normalization approaches used in well-established IQ tests [42, 43]. Normalization tables were created based on the pre-test data from participants who completed both the pre- and post-tests, including control participants who completed some amount of cognitive training during the study period. Norms were generated in 5-year age bins and tables were created within each age bin for each assessment. These normalization tables were created by taking the empirically observed percentile rank for each raw score and finding the value corresponding to that percentile from a normal distribution with a mean of 100 and standard deviation of 15 (i.e., percentile rank normalization). For the Two-Target Search and Go/No-Go assessments, where the relevant raw score was presentation time or reaction time, raw scores were reverse coded before being subjected to the normalization procedure. The resulting normalized scores compared a participant’s score to all other participants within his or her age bin, with higher scores corresponding to better performance on that assessment. The Grand Index score was calculated based on the sum of a participant’s normalized scores on all assessments. This sum was then transformed using the same percentile rank normalization procedure described above.

#### Participant-reported outcomes

Participants also completed a survey including nine questions related to specific cognitive failures [44] and successes as well as emotional status. Participants took the survey immediately after completing the neuropsychological test battery, once before beginning the study period (pre-test) and once upon completion of the study (post-test).

The survey included four questions related to a participant’s self-reported cognitive performance over the past month and an additional five questions related to a participant’s cognitive performance and emotional status over the past week. Responses to the first group of four questions rated frequency of cognitive ability or impairment, whereas the second group of five questions rated agreement or disagreement with statements about participants’ cognition or emotion. (Note: some participants were also given an additional question regarding whether they “felt benefits from cognitive training”. Because this question did not apply equally to the treatment and control groups, and was not included in the original protocol, it was removed from the analysis. For completeness, responses to this question are included along with the rest of the study data in the attached S1 Dataset). Response options for the first group of questions were: “Never”, “1–2 times during the month”, “1–2 times per week”, “Several times per week”, “Almost every day”, or “N/A”. Response options for the second group of questions were on a Likert scale: “Strongly disagree”, “Disagree”, “Neither agree nor disagree”, “Agree”, “Strongly agree”, or “N/A”. The survey items are presented in the Results section.

## Results

### Primary outcome measure

**Fig 2:**
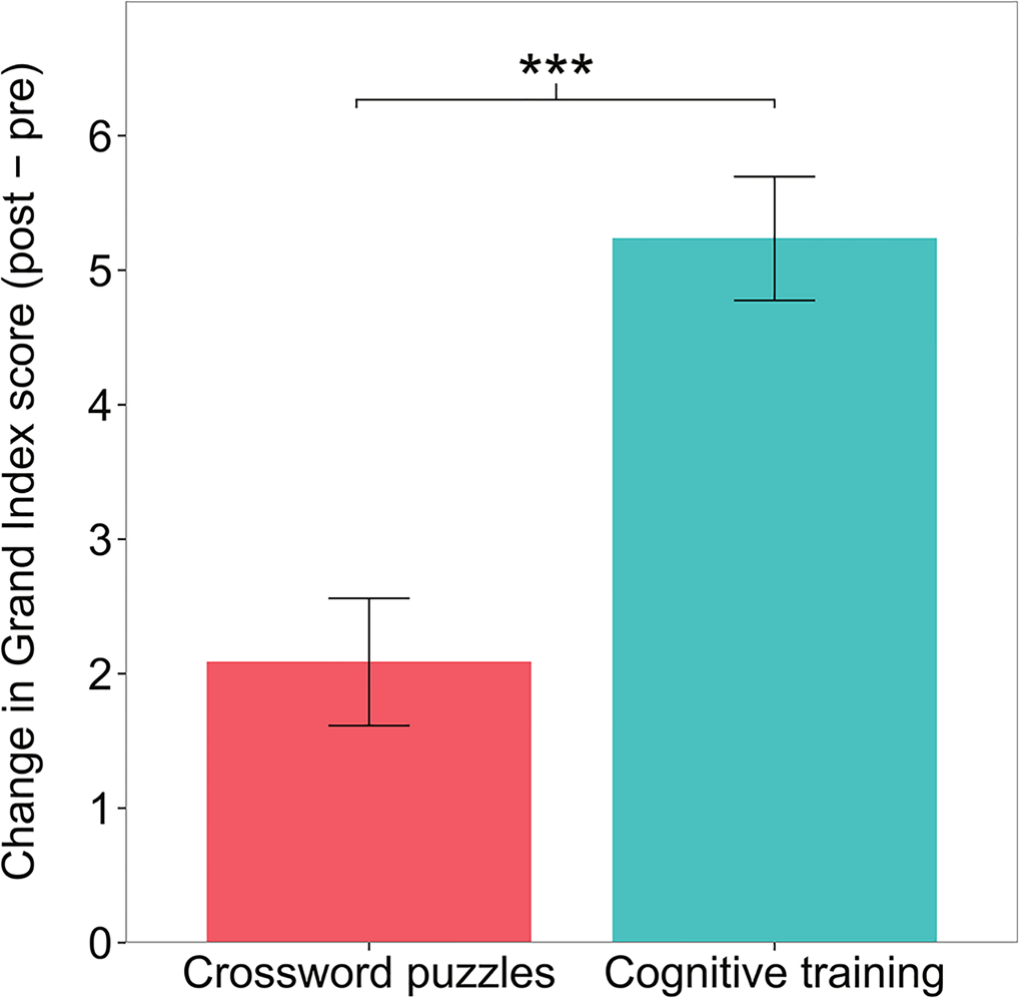
Change in composite score (Grand Index) for the cognitive training treatment and crossword puzzle control conditions. Error bars represent confidence intervals bootstrapped over 100,000 iterations. Mean change scores and error bars are based on unadjusted summary statistics. P value is based on results from the ANCOVA analysis described in Table 2. ***p < 0.001.

Our primary hypothesis was that the treatment program would lead to greater improvements in aggregate cognitive performance compared to the active control, as measured by the neuropsychological assessment battery. If this hypothesis were correct, we would expect to see larger improvements from pre-test to post-test on the Grand Index of the assessment battery for the treatment group relative to the control group. Such differences in change scores were observed. The mean increase on the Grand Index score (post-pre) in the treatment group was 5.24 points (sd = 12.00), and the mean increase in the control group was 2.09 points (sd = 10.66) (Fig 2). The 95% confidence interval for the mean difference of 3.15 points was 2.49 to 3.81.

The difference in composite (Grand Index) change scores between the two groups (treatment vs. control) was evaluated with an ANCOVA model measuring the effect of group, controlling for the pre-test score. Pre-test score was included as a covariate to control for regression to the mean effects as well as any effects of baseline performance. Cohen’s *d* effect sizes were calculated using model-adjusted means and standard deviations throughout the following analyses [45]. The ANCOVA revealed that aggregate cognitive performance improvement in the treatment group was significantly greater than in the control group (t(4712) = 8.73, p < 10^−15^, Cohen’s *d* = 0.255, 95% confidence interval = [0.198, 0.312]) (Table 2). While the improvement in the crosswords control group was also significant on a within-group basis (p < 10^−15^), the treatment group improved more than twice as much as the control. The effect size of the within-group change score was *d* = 0.467 for the treatment group and *d* = 0.212 for the controls. These results indicate that the cognitive training treatment condition was more effective than the crosswords control for improving cognitive performance on the assessment battery on an aggregate basis.

**Table 2:** Neuropsychological assessment baseline means, change scores, and effect sizes.

	Crossword Puzzles		Cognitive Training		Between-Group Difference in Change Means	
	Baseline Mean (sd)	Change Mean (sd)	Baseline Mean (sd)	Change Mean (sd)	p Value	Cohen’s *d* Effect Size (95% Confidence Interval)
Forward Memory Span	100.13 (13.74)	0.32 (15.85)	99.50 (14.16)	2.73 (16.13)	p < 10^−6^	0.152 (0.094, 0.209)
Reverse Memory Span	100.31 (13.93)	0.64 (15.84)	99.65 (13.99)	2.57 (16.39)	p = 0.0001	0.113 (0.055, 0.170)
Grammatical Reasoning	100.58 (14.53)	2.77 (13.93)	99.36 (14.78)	2.27 (14.09)	p = 0.006	-0.081 (-0.139,-.024)
Progressive Matrices	100.08 (14.49)	1.30 (15.06)	99.66 (14.90)	3.02 (15.86)	p = 0.0002	0.111 (0.053, 0.169)
Go/No Go	100.40 (14.87)	1.61 (14.95)	99.96 (15.10)	4.00 (15.73)	p < 10^−7^	0.163 (0.106, 0.221)
Arithmetic Reasoning	100.77 (14.88)	0.75 (10.32)	99.08 (14.87)	3.64 (10.78)	p < 10^−15^	0.249 (0.191, 0.306)
Two-Target Search	100.18 (14.87)	0.37 (17.92)	99.60 (15.03)	1.28 (19.23)	p = 0.27	0.032 (-0.025, 0.090)
Grand Index	100.64 (14.74)	2.09 (10.66)	99.15 (15.16)	5.24 (12.00)	p < 10^−15^	0.255 (0.198, 0.312)

Means and standard deviations of baseline and change scores are the unadjusted summary statistics. Significance levels and effect sizes are based on ANCOVA models controlling for pre-test means. For all analyses, N_control_ = 2,048, N_treatment_ = 2,667.

The model also revealed a significant negative effect of pre-test score (t(4712)) = -24.4, p < 10^−15^), indicating that, on average, participants with lower pre-test scores showed greater improvements at post-test than those with higher pre-test scores. This effect may be due to a regression-to-the-mean effect and/or an effect of starting level.

To ensure that the exclusion of control participants who did some cognitive training with the treatment program (see Participants section in Methods) could not explain these results, we performed an additional set of ANCOVA analyses (S1 File). These analyses repeated the ANCOVA approach described above, adding back in the participants from the control condition who were initially excluded because they performed some cognitive training during the study period (N = 330). The pattern of results and conclusions remained consistent across all comparisons (see S1 File), indicating that these exclusions could not explain the main result that cognitive training led to larger gains in cognitive performance compared to crosswords.

In the primary analysis conducted here, no outliers were removed. All completed assessments were included in the analysis. In order to ensure that outliers did not play an important role in the findings, we completed a secondary outlier analysis (see S1 File). In this analysis, any raw scores that were outside the range of three standard deviations above or below the mean were removed prior to further statistical analysis. The conclusions remained the same across all subtests included in the battery. The Grand Index change score analysis was recalculated for participants with no outliers. The between-group effect size for participants without outliers was Cohen’s *d* = 0.267 (95% confidence interval [0.208,0.326]). Based on this analysis, outlier effects could not account for the results of this study.

### Individual assessments

**Fig 3:**
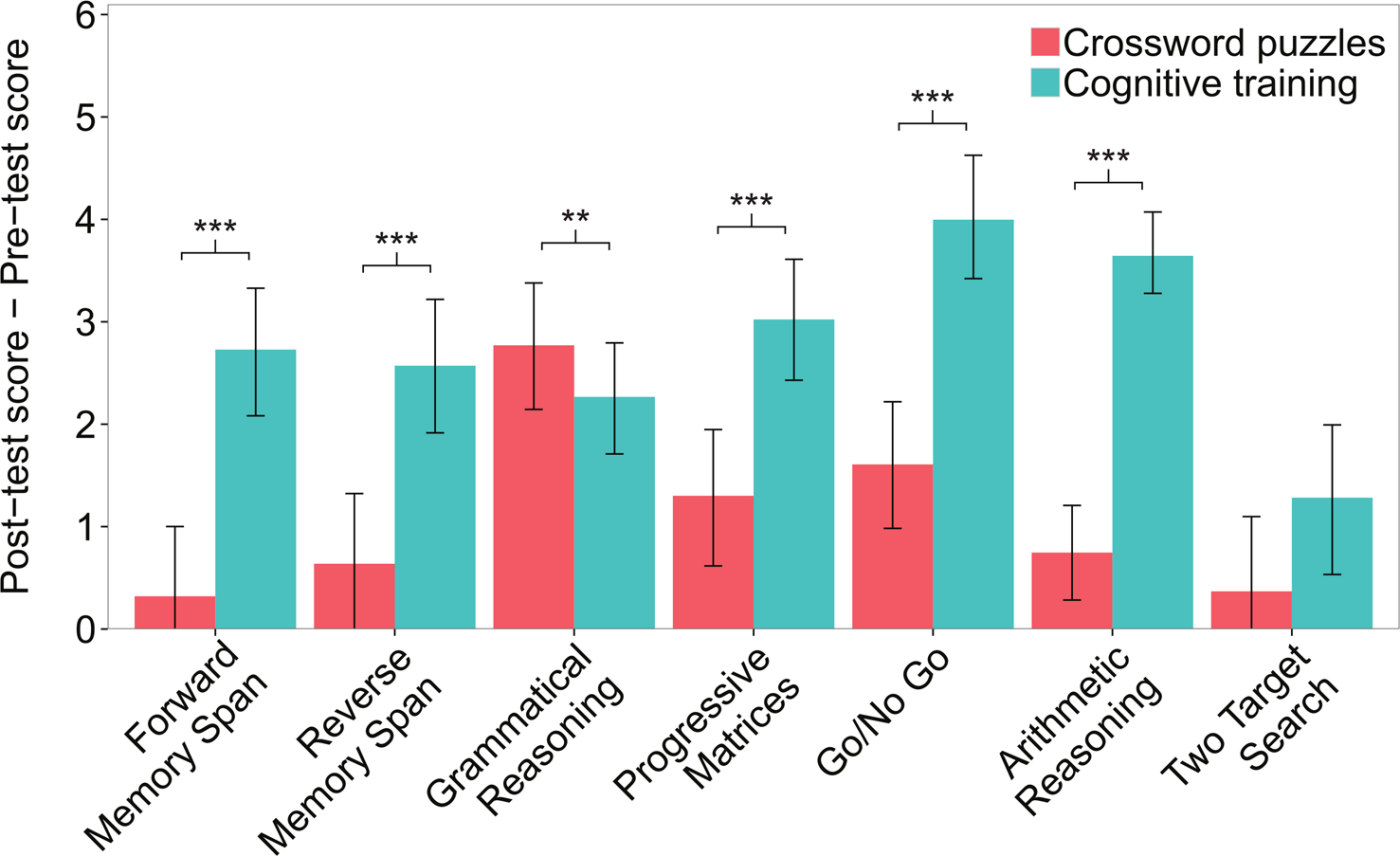
Change in individual assessments of cognitive ability. Error bars represent confidence intervals bootstrapped over 100,000 iterations. Mean change scores and error bars are based on unadjusted summary statistics. P values are based on results from the ANCOVA analyses listed in Table 2. **p < 0.01, ***p < 0.001.

Based on the significant main effect on our primary outcome measure, we performed secondary analyses consisting of additional ANCOVA models for each assessment. The models revealed that the cognitive training treatment group improved significantly more than the crossword puzzles control group on five of the seven assessments. Specifically, significantly larger improvements for the treatment relative to the control group were found for Forward and Reverse Memory Span, Progressive Matrices, Go/No Go, and Arithmetic Reasoning, while the control group improved more than the treatment group on Grammatical Reasoning. There was no statistically significant difference between the groups for the Two-Target Search task. Fig 3 provides an illustration of the unadjusted change scores for each assessment for both groups. ANCOVA model p values and effect sizes along with unadjusted pre-test means and change scores for each assessment are shown in Table 2.

### Effects of amount of engagement

**Fig 4:**
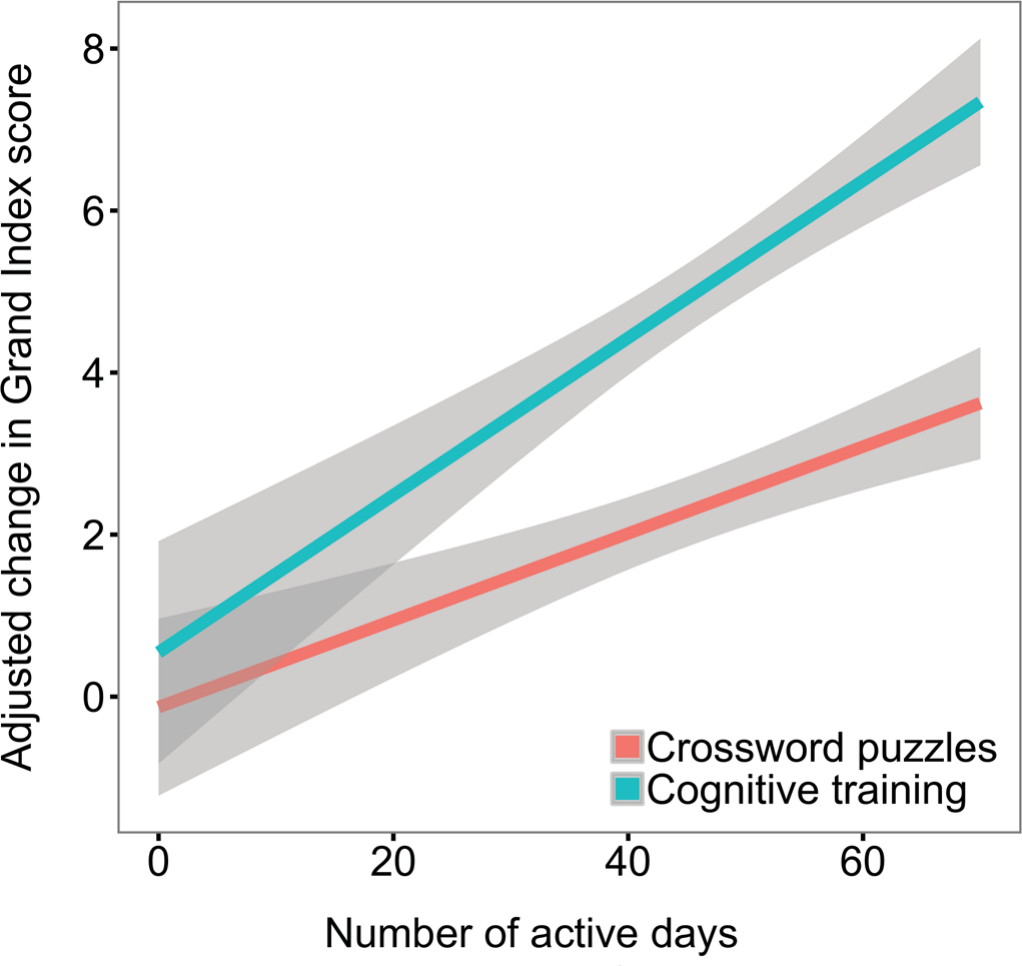
Change in composite score (Grand Index) by number of active days in treatment and control conditions. Lines represent estimates from the general linear model including effects of group, active day, and the group-by-active-days interaction. Shading represents 95% confidence intervals.

If the cognitive training treatment was more effective than playing crossword puzzles for improving cognitive abilities, we may observe a larger effect of active days of study engagement for the treatment condition compared to the control condition. The distributions of number of active days were similar for the two training conditions (treatment: mean = 46.6, sd = 15.2, median = 50; control: mean = 45.5, sd = 19.6, median = 52). Participants in the crosswords condition were active on slightly fewer days than those in the treatment condition on average (t(4713) = 2.18, p = 0.030). In order to test for a group difference in the effect of active days, we constructed a general linear model predicting Grand Index change score from pre-test score, treatment group, active days, and the group-by-active-days interaction. The model revealed both a main effect of active days (B = 0.054, t(4710) = 4.47, p < 10^−5^) and a group-by-active-days interaction (B = 0.043, t(4710) = 2.38, p = 0.017), indicating significant dose-response effects for both groups, with a significantly larger effect of dose for the cognitive training treatment group relative to controls (Fig 4).

The estimated total time participants engaged with their respective conditions provides an additional measure of compliance. Total engagement time was similar across the two groups (treatment: mean = 16.1 hrs, sd = 16.4 hrs, median = 12.2 hrs; control: mean = 13.0 hrs, sd = 7.6 hrs, median = 13.6 hrs), with mean total engagement time being higher in the treatment condition and median total engagement time being higher in the control condition. These results indicate that participants in both conditions on average complied with the instructions to engage for at least 15 minutes per day, 5 days per week for 10 weeks (12.5 hrs total). See S1 File for matched sample analyses demonstrating that the observed group differences in overall cognitive performance improvement are not explained by differences in the distributions of total engagement time.

### Participant-reported outcomes

Of the 4,715 participants included in the analyses above, 4,697 (99.8%) also completed the participant-reported outcomes survey both before and after the study period. In order to calculate change scores on the survey, participant responses were first numerically coded on a scale from 0 to 4, with the scale always ranging from 0 as the most negative response to 4 as the most positive response. Responses to questions 1, 2, 3, 7, 8, and 9 were reverse coded to maintain consistency of response coding across all questions (i.e., such that a higher number indicated a more positive response). An average of the scores was taken for both pre- and post-tests as an overall measure of self-reported real-world cognitive performance and emotional status. The differences between pre- and post-test overall scores and scores on each question were analyzed.

The hypothesis that participants in the treatment group would show greater self-reported improvements in cognition and emotional status relative to control participants was tested via an ANCOVA model measuring the effect of group (treatment vs. control) on the change in average survey score, controlling for average pre-test score. The model revealed a main effect of group on change score, indicating that cognitive training resulted in larger increases in self-reported cognition and emotional status scores compared to the crossword puzzles control (t(4689) = 8.50, p < 10^−15^, Cohen’s *d* = 0.249, 95% confidence interval = [0.191, 0.306]). The improvement in the crosswords control group was also significant on a within-group basis (p < 10^−15^). These results indicate that, overall, the cognitive training treatment was more effective than the crosswords control for improving self-reported real-world cognition and emotional status. Pre-test score was a significant negative predictor of post-test score (t(4689) = -39.8, p < 10^−15^), indicating regression to the mean and/or effect of starting level.

For all nine questions, both groups tended to report improvements following study participation, compared to the pre-test. The changes were significant for both groups on all questions except for question 4 (memory for a new name). Results for each question are presented in Table 3. Participants in the treatment group reported significantly larger improvements for eight of the nine questions compared to the control group (all ps<0.01). The three largest group differences were on questions 1, 3 and 6, all of which were related to concentration.

**Table 3:** Participant-reported outcome questions and results.

		Crossword Puzzles		Cognitive Training		Between-Group Difference in Change Means		
	During the past month, how often have you…	Baseline Mean (sd)	Change Mean (sd)	BaselineMean (sd)	Change Mean (sd)	N_control_, N_treatment_	p Value	Cohen’s *d* Effect Size (95% Confidence Interval)
1	… lost track of details as you were reading and needed to go back and re-read sections?	2.89 (1.16)	0.48 (1.12)	2.92 (1.14)	0.64 (1.15)	1,984, 2,586	p < 10^−8^	0.180 (0.122, 0.239)
2	… misplaced items (e.g., reading glasses, keys) around the house?	3.19 (1.16)	0.47 (1.01)	3.27 (1.14)	0.52 (1.05)	2,009, 2,617	p = 0.0004	0.105 (0.047, 0.163)
3	… found yourself losing concentration during a conversation?	3.10 (1.21)	0.46 (1.14)	3.14 (1.22)	0.62 (1.16)	1,976, 2,592	p < 10^−9^	0.186 (0.128, 0.245)
4	… remembered someone's name who had just been introduced to you?	3.01 (1.20)	0.04 (1.41)	2.97 (1.21)	0.13 (1.46)	1,727, 2,179	p = 0.07	0.058 (-0.005, 0.121)
	Rate your experience over the LAST WEEK…							
5	I felt creative.	3.48 (1.07)	0.15 (1.08)	3.54 (1.04)	0.20 (1.05)	2,004, 2,589	p = 0.001	0.096 (0.038, 0.155)
6	My ability to concentrate was good.	3.30 (0.99)	0.41 (1.07)	3.34 (0.99)	0.58 (1.09)	2,015, 2,616	p < 10^−14^	0.232 (0.174, 0.289)
7	I felt anxious.	2.59 (1.17)	0.34 (1.28)	2.61 (1.16)	0.49 (1.27)	1,990, 2,574	p < 10^−6^	0.154 (0.096, 0.213)
8	I was in a bad mood.	2.90 (1.16)	0.28 (1.22)	2.94 (1.13)	0.38 (1.20)	1,989, 2,565	p < 10^−4^	0.119 (0.060, 0.177)
9	I felt sad for no obvious reason.	3.37 (1.21)	0.27 (1.25)	3.33 (1.21)	0.40 (1.24)	1,964, 2,537	p = 0.001	0.096 (0.038, 0.155)
	Overall Average	3.10 (0.60)	0.33 (0.57)	3.12 (0.59)	0.44 (0.60)	2,038, 2,654	p < 10^−15^	0.249 (0.191, 0.306)

Means for questions 1, 2, 3, 7, 8 and 9 were reversed coded, such that higher scores should be interpreted as more positive responses for all questions. Means and standard deviations of baseline and change scores are the unadjusted summary statistics. Significance levels and effect sizes are based on ANCOVA models controlling for pre-test means. Degrees of freedom are based on participants giving non-“NA” answers at both time points.

## Discussion

The findings of this study are consistent with the extant literature on cognitive training that shows that progressively challenging, targeted cognitive training can be an effective tool for improving core cognitive abilities including speed of processing [13], working memory [46], and fluid reasoning [10]. The results presented here extend previous findings by demonstrating that a cognitive training program targeting a variety of cognitive capacities with different exercises can be more effective than crossword puzzles at improving a broad range of cognitive abilities.

After 10 weeks of training, participants receiving the cognitive training treatment improved more than those receiving crossword puzzles on Forward and Reverse Memory Span (measures of visual short term and working memory, respectively), Progressive Matrices (a measure of fluid reasoning), Go/No-Go (a measure of response inhibition and speed of processing), and Arithmetic Reasoning (a measure of problem solving). In addition, improvement on the overall measure of cognitive function used as the primary outcome measure in this study–the Grand Index for the assessment battery–was more than twice as large in the cognitive training group as it was in the crossword puzzles control group. Thus, for improving a variety of core cognitive abilities, the treatment used in this trial was more effective than crossword puzzles.

The Cohen’s *d* effect size for the between-group differences in the primary outcome measure (the Grand Index) was 0.255. Another approach to appreciating the magnitude of these results is to contextualize them in the distribution of scores on the outcome measures. We observe that participants in the training group improve by 2.77 points more than those in the crosswords group, after correcting for the pre-test score in our ANCOVA model. Given that the scores are scaled on a 100 mean ± 15 sd scale, we can evaluate how far an average participant would move within the population distribution (for their age) based on moving a given number of points. In this case, 2.77 points is the equivalent of moving from the 50^th^ percentile of the distribution to the 57^th^ percentile. This is a potentially meaningful move within the distribution.

A significant group-by-active-days interaction was observed in this study, such that an additional active day engaging with the cognitive training intervention was related to larger gains on the cognitive battery composite score compared to an additional active day engaging with crossword puzzles (Fig 4). This suggests that additional training could lead to larger gains. While it is unlikely that the linear relation holds indefinitely (i.e., the function likely decelerates at some point), future work will be necessary to ascertain how much total improvement is possible over longer training periods.

In addition to the enhanced performance observed in the cognitive training group on the neuropsychological measures of cognitive function, participants in this group also self-reported experiencing benefits that were significantly greater than those reported by participants in the active control. These participant-reported improvements were particularly strong on questions related to the ability to concentrate. These results suggest that participants in the treatment group experienced benefits from the training in their everyday lives.

Crossword puzzles were chosen as the active control because they are commonly believed to be a cognitively stimulating activity that is good for brain health [31, 32]. This is important because it has been suggested that belief in the efficacy of a training intervention could affect effort and performance on testing outcomes [47].

While not as large as the gains seen in the treatment group, participants in the crosswords control group also showed improvements in cognitive performance. Without a no-contact control group in this study, it is not possible to conclusively determine whether these improvements in the active control condition were due to practice effects, placebo effects, real treatment effects, or some combination of these. Further study will be needed to better understand the benefits of crossword puzzles for maintenance and enhancement of cognition. It is worth noting that participants in the crosswords group improved slightly more than the cognitive training group on a measure of grammatical reasoning. It would not be unreasonable to imagine that intense, concerted word finding training might enhance one’s performance on a linguistic task.

There are several reasons why the treatment program might have outperformed crossword puzzles in enhancing cognitive function. First, the cognitive training program is specifically *targeted* to core cognitive functions. This distinguishes the treatment from crossword puzzles, which are not designed with the goal of cognitive enhancement. Another central feature of the cognitive training program studied here is that it is *progressively challenging*–that is, many of the tasks explicitly increase in difficulty as the individual improves, while others encourage the individual to perform at threshold by rewarding increasingly faster and more accurate performance (see S1 Appendix). This follows a long-established tenet in the psychological literature, that learning conditions are optimized when the task is challenging, but not prohibitively difficult [48, 49]. Task variety and novelty are also potentially important. In the case of crossword puzzles, participants are primarily involved in vocabulary retrieval, challenging a more limited set of neural pathways. In the cognitive training program studied here, participants are challenged to engage with a variety of cognitive tasks that challenge different neural processing systems and do so in different ways. This variety limits the opportunity to solve the tasks with a single task-specific strategy, thus encouraging the learning of new strategies and the development of new neural connections.

We noted that there have been several studies that have reported not finding benefits from cognitive training. The only other similarly powered study that did not find positive results is a 2010 study that recruited 11,430 participants through a BBC television show and collected data online [17]. The authors concluded that brain training had no measureable benefits. Several key aspects of that study differ from the one presented here. First, neither of the two treatment conditions they used had been studied empirically prior to that experiment. As we demonstrate in this study, not all cognitively stimulating activities are equally effective for enhancing cognition, and it is possible that other programs not examined in their study are more effective. Also, the average amount of training exposure in the BBC study was less than half of that in this study. This is an important distinction as results of this study indicate that amount of training is related to the magnitude of gains in cognitive performance (Fig 4).

Our results represent statistically significant improvements in cognitive processes through training. This study included a sufficiently large number of participants and enough training to reliably detect these effects. As has been noted previously [50], most cognitive training studies that have shown null results have not been powered in such a way that either a positive or a null outcome would be informative, and often include quite short training periods. In the broader context of factors influencing cognitive processes (i.e., a lifetime of experiences), even the 10 weeks of training in this study is a fairly modest amount. Further research will be needed to understand how the current effects extrapolate over much longer training periods.

In this study, 49.1% of participants randomized into one of the two conditions were lost to follow-up (i.e., did not take the post-test). There was little difference in dropout rates between the two groups, and supplemental analyses (S1 File) that equated the engagement characteristics of completers from both groups demonstrated that these differences in dropout between the two groups could not explain the results.

This study utilized an entirely online design. The online methodology is ecologically valid, since most users in the real world experience the program at home or in some other personal environment outside a laboratory or clinic. In traditional laboratory-based training studies, participants experience considerable contact with study personnel. Contact with study personnel may lead to lower rates of loss to follow up. However, this personal interaction introduces a variable that could affect the results and is not reflective of how most normal, healthy adults use these programs.

A possible limitation of the current study is that it does not isolate specific mechanisms. For example, adaptive difficulty may play an important role in driving the transfer of training [51]. Many of the tasks in the cognitive training program were explicitly adaptive (i.e., difficulty was increased as performance improved), whereas the crossword puzzles were not. The two conditions differed on other dimensions as well. The cognitive training program incorporated a variety of tasks that targeted specific cognitive functions, while the crosswords condition did not. Future research is needed to more fully elucidate the relative contributions of particular components of activities that lead to improvements in cognitive performance.

Future studies could also extend the cognitive domains tested. Our neuropsychological assessment battery was relatively comprehensive across a variety of domains, but not every possible dimension of cognition was addressed. For example, this battery did not include any assessment explicitly targeting learning (e.g., Hopkins Verbal Learning Test) or complex working memory (e.g., Counting Span).

Research on training to improve cognitive skills is not complete, and there remain many open questions. The ability to efficiently collect large data sets in controlled experiments over the Internet may prove crucial to answering the open questions related to cognitive training in the future.

## Supporting Information

S1 CONSORT Checklist Completed CONSORT 2010 checklist of information to include when reporting a randomized trial. (PDF)

S1 Appendix Descriptions of each of the cognitive training tasks used in this study. (DOCX)

S2 Appendix A more thorough description of the seven neuropsychological assessments used to measure cognitive performance at pre-test and post-test. (DOCX)

S1 Dataset The study data for the statistical comparisons of outcomes for cognitive training relative to crosswords. (CSV)

S2 Dataset A description of the data columns in S1 Dataset. (CSV)

S1 File Additional Analyses. The first supplementary analysis is an ANCOVA analysis that includes participants assigned to the control group who engaged in some cognitive training during the study period. The second supplementary analysis describes how engagement time is estimated in the two conditions and provides a paired-matching analysis that controls for the total time spent engaging with the two conditions. The third supplementary analysis includes an outlier removal procedure.
(DOCX)

S1 Protocol The IRB-approved study protocol. (PDF)
