# Supplementary material for: Enhancing Cognitive Abilities with Comprehensive Training: A Large, Online, Randomized, Active-Controlled Trial
Source: PLoS One. 2015 Sep 2;10(9):e0134467. doi: 10.1371/journal.pone.0134467 (PMC4557999; doi:10.1371/journal.pone.0134467)
Supplement: S1 Appendix — (DOCX) [file pone.0134467.s002.docx]

**S1 Appendix.** Descriptions of each the cognitive training tasks used in this study.

**Addition Storm**

Addition Storm is similar to Raindrops (see below), but restricted to addition problems.

**Birdwatching**

Birdwatching engages and exercises distributed and divided visual attention and speed of processing by challenging the user to identify a centrally presented letter while simultaneously indicating the position of a peripherally presented bird. The visual system is challenged further by the introduction of complex backgrounds, picturing natural scenes. In addition to the visual challenge, the user is rewarded by engaging in a secondary task in which a word must be constructed from the identified letters. The game advances to the next level when the user achieves a criterion level of performance. Each progressive level is marked by shorter presentations of central and peripheral targets and increasing numbers of distractor objects in the periphery.

**Brain Shift**

Brain Shift requires the user to switch rapidly between two different tasks. Two cards are presented on the screen. On each trial, a number and a letter appear on one of the cards. If the stimulus appears on the top card, then the user should indicate “yes” if the number is even and “no” if the number is odd. On the other hand, if the stimulus appears on the bottom card, the user should indicate “yes” if the letter is a vowel and “no” if the letter is a consonant. After a number of correct responses, the challenge is increased by removing the prompt explaining the rules; thus, the user must remember the rules while accurately and rapidly indicating responses. The goal is to make as many correct responses as possible in a fixed period of time.

**Brain Shift Overdrive**

Brain Shift Overdrive is very similar to Brain Shift (see above), but with increased challenge in both task switching and working memory. In this version, there are four cards, arranged in a grid. If the number and letter appear in the upper left, then the correct response is to click “yes” when the number is even and “no” when the number is odd. If the number and letter appear in the lower left, then the correct response is to click “yes” when the number is odd and “no” when the number is even. The user is looking for a vowel in the upper right and a consonant in the lower right.

**By the Rules**

In By the Rules, users must identify the hidden rule in a dynamic card game by indicating whether each revealed card follows the rule. Rules involve the characteristics of the pattern depicted on the card, such as color, shape, number, and pattern. Exercising mental flexibility and working memory, the user is challenged to formulate hypotheses about what the current rule might be and then dynamically update that hypothesis as new information becomes available. The user is told when they have correctly guessed a particular rule and when the next rule is being presented. This type of reasoning ability involves the intersection of inductive and deductive reasoning, and in that way mimics the type of decision-making that happens in a wide variety of real world contexts.

**Chalkboard Challenge**

In Chalkboard Challenge, users are presented with two arithmetic expressions, one on the left and one on the right, and must indicate which expression is larger (or whether they are equal). The game rewards speed. As users complete trials successfully, the arithmetic expressions become more complex, beginning with single numbers and progressing up to expressions including several integers, operators (addition, subtraction, multiplication, and division), and levels of parentheses.

**Color Match**

Color Match is based on the Stroop Task, a classic test of semantic interference between color and meaning of words. Each trial shows two cards, and each card displays a color word (black, red, blue, or yellow). Additionally, the text displayed on the right card is colored with one of the four aforementioned colors. Users must read the word on the left card and indicate “yes” if the meaning of that word matches the color of the text on the right card and “no” if the color does not match. The user should ignore the meaning of the word displayed on the right card and only pay attention to its color. The goal is to make as many correct responses as possible per 45 second session, and users are additionally rewarded for completing sequential trials correctly.

**Disconnection**

Disconnection is similar to Disillusion (see below) except that the colors and shapes change in each session.

**Disillusion**

Disillusion requires the user to switch rapidly between two sub-tasks. Users are presented with a grid of puzzle pieces, each of which is marked with a colored shape. An additional piece is presented and users must place it on the puzzle. The orientation of that piece dictates whether to match based on the color attribute (if long dimension is along vertical axis) or shape attribute (if long dimension is along horizontal axis). Users must learn the association and then place the pieces as quickly as possible before a time limit in order to progress.

**Division Storm**

Division Storm is similar to Raindrops (see below), but is restricted to division problems.

**Eagle Eye**

Eagle Eye is similar to Birdwatching (see above), but the task adapts within a session to the user’s performance. The user must identify a centrally presented number while simultaneously indicating the position of a peripherally presented bird. The game increases in difficulty by shortening presentation time and increasing visual range.

**Ebb and Flow**

Each trial consists of a screen full of leaves all pointing in one direction and traveling in the same or a different direction. The leaves are color coded and depending on the color, the user’s task is either to indicate the direction the leaves are pointing or the direction the leaves are moving. The response is a 4-alternative forced choice task (up, down, left, or right). Trials can either be congruous, in which the leaves are pointing in the same direction that they are moving, or incongruous, in which the leaves are pointing in a different direction from the direction of motion. The color of the leaves, that is, the rule that the user is to follow, is maintained for a random number of trials in order to build up an automatic response. The rule is then switched, and users must then switch their focus to the opposing attribute. This task exercises response inhibition and task switching.

**Face Memory Workout**

Face Memory Workout is similar to Memory Match (see below). Rather than matching abstract objects, however, users must recognize and match human faces. Users can progress from 1-back to 2-back to 3-back, meaning that users must match the current trial with the trial that appears one trial previously, two trials previously, or three trials previously.

**Familiar Faces**

Familiar Faces challenges the user’s ability to create associations between visual and verbal information, such as associating a person’s name with their face. The user’s job in this game is to work as a restaurant server. The user is presented with visitors, who have names and place orders. The user must remember the orders as well as the customers’ names to earn large tips.

This game is an exercise of associative memory that is closely related to the kinds of memory challenges that are experienced on a daily basis. As performance improves on this task, it becomes more complicated, with more characters and more complex orders. Not only does the user need to remember names during a single session, the user must also remember names from past sessions, mimicking the real life situation.

**Follow That Frog**

Follow That Frog is a spatial working memory task that challenges users to follow the hops of a frog across a pond filled with lily pads. The game is an adaptive spatial version of the n-back task where users must indicate where the frog hopped a given number of hops back. This number increases with several successive successful trials and decreases after a mistake. Progress is persisted across sessions.

This game is a variation on n-back designed to be more interactive than the standard match/no match framework. The basic premise is a follow-the-leader task, where the user must follow the computer-controlled frog and jump on the same lily pads in the same order. In this variation, the n value is the number of moves behind the user is from the computer-directed frog–and the number of steps ahead the user must remember. N-back tasks exercise working memory and updating.

**Lost in Migration**

Lost in Migration utilizes a simple flanker task to exercise focus of attention and response inhibition. Each trial consists of a presentation of five birds pictured in a pattern resembling a flock in flight. The user’s task is to indicate the direction of the middle bird. The response is a 4-alternative forced choice task. Trials can be either congruous, where the middle bird is pointed in the same direction as the other birds, or incongruous, where the middle bird is pointed in a different direction. The challenge of response inhibition occurs on the incongruous trials, when the user is tempted to indicate the direction of the majority of birds. The goal is to make as many correct responses as possible per 45 second session.

**Memory Lane**

Memory Lane is a stylized version of the challenging dual n-back task. The user is presented with successive apartment buildings passing by. As an apartment passes, a person appears at a window and speaks a letter. Users must remember which window location appeared and which letter was spoken a set number of apartments previously. Users compare the previous location and letter to a current location and letter, indicating a location match by pressing the left arrow, a letter match by pressing the right arrow, and withholding a press to indicate the lack of match. The game challenges memory further by evaluating whether users recall all letters that they saw during the round.

**Memory Match**

Memory Match is a 2-back visual working memory task. Users indicate whether the current symbol matches the one presented two symbols previously. A symbol is presented in a box on the right of the screen. The user indicates whether there is a 2-back match, and then the symbol shifts one position to the left. After the next response, the first symbol slides one more position to the left and is now the to-be-matched target. Initially, the current symbol and the previous two are all visible.

**Memory Match Overload**

Memory Match Overload is similar to Memory Match (see above), but is significantly more challenging because the task is 3-back. In other words, users must indicate whether the current symbol matches the one presented 3 symbols previously.

**Memory Matrix**

In Memory Matrix, users exercise their spatial short-term memory by remembering the location of squares on a grid. The target squares appear briefly and simultaneously, and the user must remember the location of all of the target squares and click on them at the end of the presentation. Initially, three squares appear and the grid size is 3×3.  However, with each correct response, the number of targets is increased by one, and the grid size grows as well. Each time an incorrect response is made, the number of targets reduced by one and the grid size shrinks.

**Moneycomb**

Moneycomb is a spatial memory task in which users must remember the locations of coins hidden within a hexagonal grid (honeycomb) structure. Groups of copper, silver, and gold coins labeled with values 1, 2, and 3, respectively appear in sequence. Users must recall the locations of these coins in the order of their value. Difficulty increases considerably by increasing the number of coins.

**Monster Garden**

Monster Garden is a visuospatial working memory exercise that challenges the user to remember the location of several obstacles that appear only momentarily. The user has the additional challenge of maintaining the locations of the obstacles in working memory while navigating the game environment. The context of the game is a garden scene that is divided into a grid pattern. Initially, obstacles in the form of cartoon monsters appear briefly in various squares throughout the grid. The obstacles are presented such that only one is visible at a time. Next, a goal in the form of a flower appears in one of the squares. The flower stays visible while the user attempts to navigate the character through the garden by stepping only on open squares and avoiding squares where the no-longer-visible obstacles are. If the character steps on an obstacle, a life is lost. The game ends after 3 lives are lost. Levels increase in difficulty by expanding the grid and increasing the number of obstacles that the user must maintain in working memory.

**Multiplication Storm**

Multiplication Storm is similar to Raindrops (see below), but restricted to multiplication problems.

**Name Tag**

In Name Tag, users must discover and remember matching pairs of cards within a grid. At the beginning of each session, name tags are displayed that include names paired with the corresponding faces. During game play, users must match name cards with their corresponding face cards.

**Observation Tower**

Observation Tower challenges users to process briefly flashed information quickly. In the task, a set of numbers is presented for a short time and then disappears, leaving only indicators of the numbers’ locations. Users must indicate the order of digits they saw in ascending order. Each digit observed and correctly ordered goes towards constructing a tower. The goal is to recall and correctly order as many digits as possible. Observation Tower adapts to user performance by decreasing presentation time as well as increasing the number of digits to remember as the user demonstrates proficiency.

**Penguin Pursuit**

This game targets spatial orientation by requiring users to guide a penguin through a maze. The maze repeatedly rotates its orientation so that users must transform directions in their minds – for example, up becomes left – in order to input the true directions and guide the penguin in a race against a computer-controlled penguin. The task increases in difficulty by increasing rotation frequency, increasing maze size, and decreasing the maximum time allowed.

**Pet Detective**

Pet Detective exercises users’ route planning skills. The game engages users in a task where they must move objects (pets) from one point to another. These objects are placed within a connected map. Users are limited in the distances they can travel and number of objects they can move simultaneously, and thus must plan a route several steps ahead of time to successfully complete the task. The game rewards efficiency and speed, and increases in difficulty by increasing the number of objects and map size, and dispersing the objects more.

**Pinball Recall**

Pinball Recall exercises spatial working memory by requiring the user to remember the location of multiple objects and mentally predict a ball’s path accounting for those objects. The game presents the user with a grid and one or more bumpers that have a specific orientation (±45°). The bumpers disappear after a brief presentation time (~1 second), after which a point on the outside of the grid is highlighted. The user must then determine the path a ball will travel after emanating from the highlighted point, taking the various bumpers into account (balls ricochet at 90° off bumpers), and indicate which point on the outside of the grid the ball will emerge. Difficulty increases with larger grids and more bumpers.

**Playing Koi**

Playing Koi challenges visual divided attention and working memory. The goal of the task is to feed all the koi, while avoiding the other fish. Each koi fish looks exactly the same and is moving around, and the user must feed each exactly once, which challenges the user to focus on multiple targets simultaneously and follow them throughout their journey across the screen. Focus is critical to successfully identify all of the koi exactly once and avoid the distractions of other fish moving around the screen. As performance improves, the task becomes significantly more difficult with more fish, more distractions, and fewer permitted mistakes.

**Raindrops**

Raindrops challenges basic arithmetic skills and divided attention in a speeded manner. Arithmetic problems (enclosed in water droplets) fall from the top of the screen and users must enter the answers before the problems reach the bottom of the screen. The difficulty increases considerably as the user progresses. In particular, the arithmetic problems fall with greater frequency and more complexity – the game progresses from simple single-digit addition to multiple digit addition, subtraction, multiplication, and division.

**Rhyme Workout**

Rhyme Workout is an n-back task that is similar to Memory Match (see above). Rather than matching exact objects, however, cards with words on them appear, and users must decide whether the word n trials ago rhymes with the current word. Thus, the task engages users’ phonemic recognition while simultaneously challenging working memory. Users can progress from 1-back to 2-back to 3-back.

**Rhythm Revolution**

A record is presented, around which note cues are arranged in a rhythmic fashion. The record rotates, and the user listens to the rhythm for two revolutions. Following this, the user repeats the rhythm with the spacebar for four revolutions. As the user graduates to harder trials, eighth notes, sixteenth notes, triples, and fading notes are incorporated.

**Robot Factory**

In Robot Factory, users exercise response inhibition in a task where they must respond to the appearance of robot parts on three pedestals. The task rewards speed; however, some parts that appear will not fit on the robot, and the pedestal indicates the improper part. Users must withhold their response and not select that pedestal until the part is removed. The game adapts in real time, and as users progress, parts are introduced more quickly, and more improper parts are introduced.

**Rotation Matrix**

Rotation Matrix is similar to Memory Matrix (see above), with the addition of a rotation component that makes the task more challenging. In Rotation Matrix, users exercise their spatial working memory by remembering the location of squares on a grid. The target squares are presented simultaneously for a brief period of time, and after they disappear, the entire matrix rotates 90 degrees left or right. The user must indicate the location of the target squares in their new locations, which requires them to project how the target squares would have moved during the rotation. The task begins with a 3×3 grid and one target square for the user to remember. The grid size and number of target squares increase with correct performance and decrease with incorrect performance.

**Route to Sprout**

Route to Sprout exercises planning through a series of puzzles where users must move an object (a seed) to the goal. Waypoints are connected by paths, but many waypoints contain obstacles (ladybugs). The user can move objects from waypoint to waypoint, but each waypoint can only hold one object at a time. Levels involve a connected graph that includes stack and queue structures. Users must move the objects one at a time in order to get their seed to the goal, where it is planted. The goal is to complete the puzzle in the smallest number of moves, as quickly as possible. Route to Sprout increases in difficulty by increasing path complexity.

**Space Junk**

Space Junk is a subitizing task where users are briefly presented with a number of space-themed silhouettes scattered on a black background. The silhouettes disappear and users must indicate how many objects they saw. In this task, the user generally does not have enough time to count the objects on the screen, and must instead automatically judge how many there were. Difficulty increases by increasing the number of objects and the amount of motion.

**Speed Match**

In Speed Match, a stack of cards with shapes on them is flipped one at a time, and the user indicates whether the current card matches the previous card (1-back). Performance is measured by how fast and accurate a user can perform in a fixed timeframe, and the experience is designed such that successful users will increase speed across sessions while maintaining high accuracy.

**Spatial Speed Match**

Spatial Speed Match is similar to Speed Match (see above), except that the stimuli on the cards are three circles arranged in a triangle with one circle filled, rather than the shapes used in Speed Match. This challenges spatial discrimination rather than object discrimination.

**Speech Match Overdrive**

Speed Match Overdrive is an advanced version of Speed Match. In Speed Match Overdrive, symbols have shape and color attributes, and the user indicates whether the current symbol matches the previous symbol exactly, partially, or does not match. This task challenges the user to respond quickly yet accurately. The response is biased toward accuracy by including increasing bonuses for increasingly long chains of correct responses.

**Speed Pack**

Speed Pack exercises visualization skills in a physically intuitive task. Players are presented with suitcases filled with objects. One of these objects (a camera) is moveable, and users must position that object such that when the suitcase is folded, none of the objects overlap each other. Trial complexity is adapted based on performance – more objects and more folds are introduced to users completing trials quickly and accurately. Players are encouraged to complete as many suitcases as possible within a limited amount of time.

**Star Search**

Star Search challenges users to focus their attention and find the single, unique object among a field of objects. The objects are primitive shapes with physical characteristics such as color, orientation, motion, and shading. Sets of other objects share characteristics with the target object to distract users. If users discover the unique shape quickly, the game increases in difficulty by introducing characteristics that are harder to tell apart. For example, the game begins with different shapes with different colors. At higher levels, the shapes and colors are identical, but the target object rotates in a different direction from that of the distractor objects. The number of objects that share some, but not all, characteristics also increases.

**Subtraction Storm**

Subtraction Storm is similar to Raindrops (see above), but restricted to subtraction problems.

**Tidal Treasures**

In Tidal Treasures, an increasing number of objects wash up on a beach, and in each trial the user must select an object not previously selected. Items include things like seashells, kelp, flotsam, and jetsam that one might expect to find on a beach More items appear in each trial, taxing the user’s memory, until the user selects an object that had been previously selected. As users progress through rounds, difficulty also increases by decreasing the uniqueness and distinctness of objects. The task differs from other working memory exercises by allowing users to choose the objects they wish to remember, which engages other executive functions.

**Top Chimp**

Top Chimp challenges users to process briefly presented information quickly. In the task, the user briefly sees scattered poker-style chips with numbers on them. The numbers disappear and the user must order the chips in ascending order. Users compete against computer opponents and gauge their own ability by betting a number of tokens relating to their confidence in beating the opponent. Difficulty increases in future sessions by decreasing presentation time and increasing the number of items that have to be remembered as well as the amount needed to bet in order to beat the computer opponent.

**Train of Thought**

Train of Thought engages users’ divided and distributed attention in a sustained manner by requiring them to a direct a set of color-coded trains to their destinations. Trains emerge from a tunnel and travel continuously along a branching set of tracks. Users must click switches at the branch points in order to correctly direct the trains to their destinations. Train frequency changes within a session to match a user’s performance, increasing with correct deliveries and decreasing with incorrect deliveries. When users perform well, delivering nearly all trains to their correct destination, the number of stations increases.

**Trouble Brewing**

In Trouble Brewing, players manage a coffee shop. Coffee orders are introduced and must be fulfilled by adding the requested ingredients to a cup and then starting the coffee dispenser. Care must be taken to stop the machines at the right time so that the cups are filled, but not overfilled. Players must plan out their strategy, using multiple machines, to make use of their time most efficiently, in order to serve all customers as quickly as possible. At higher levels, players must manage multiple screens, taking orders on one, and executing them on another.

**Word Bubbles**

Word Bubbles is designed to exercise verbal fluency. A three-letter word stem is presented and users are given 1 minute to enter as many words as possible that begin with that stem. To receive high scores, users must enter words of various lengths from 4 letters to 13+ letters.

**Word Bubbles Rising**

Word Bubbles Rising enhances the gameplay of Word Bubbles (see above) by introducing 4 and 5 letter word stems in subsequent rounds, which are shortened to 3 letters after a set time interval.

**Word Sort**

Word Sort is similar to By the Rules (see above). However, instead of pictorial items, words must be classified. Word categories can include the number of letters, typeface, starting and ending letter, and semantic relationships.
