# Supplementary material for: Enhancing Cognitive Abilities with Comprehensive Training: A Large, Online, Randomized, Active-Controlled Trial
Source: PLoS One. 2015 Sep 2;10(9):e0134467. doi: 10.1371/journal.pone.0134467 (PMC4557999; doi:10.1371/journal.pone.0134467)
Supplement: S2 Appendix — (DOCX) [file pone.0134467.s003.docx]

**S2 Appendix.** A more thorough description of the seven neuropsychological assessments used to measure cognitive performance at pre-test and post-test.

**Descriptions of Assessments Used in Primary Outcome Battery**

These assessments feature a white background with black text, and fill a window on the computer screen 640 pixels in width by 480 pixels in height, unless otherwise noted. Each assessment is introduced through text directions as well as two trials of interactive practice at the lowest difficulty level. Practice trials are repeated until the participant correctly completes the trials, ensuring that the participant understands the task. Feedback regarding correctness is given during practice trials, but is not given during regular trials of the assessment, unless otherwise noted.

**Forward Memory Span**

This assessment is based on the Corsi Blocks tasks [1]. Blue circles with radii equal to 1/20 of the window height are placed at randomized, non-overlapping spatial locations and individually highlighted in orange following a particular sequence. Circles are highlighted for 500 msec with a 100 msec inter-stimulus interval. The participant is asked to recall the sequence by clicking on each circle in same order as originally presented. The length of the sequence increases by one every two trials. The session ends when the participant gives two incorrect answers at the same span level. The total number of correct responses is the dependent measure. This task is considered a measure of visual short-term memory.

**Reverse Memory Span**

This assessment is identical to the forward visual memory span assessment, except that the participant is asked to recall the sequence of circles in the reverse order. It is considered a measure of visual working memory.

**Grammatical Reasoning**

The Grammatical Reasoning assessment [2] measures the participant’s facility with rapidly and accurately evaluating a potentially confusing grammatical statement. A blue square and a blue equilateral triangle, each with height equal to 1/5 of the window height are shown side by side, with a logical statement written below. For example, the square may be positioned to the left of the triangle on a particular trial. The participant could be prompted with a statement of the form, “The square is not to the left of the triangle.” In this case, the answer would be “false.” The participant responds whether the statement is true or false by pressing a key on the keyboard that is indicated as corresponding to true or false. The probability that the statement includes a negative (“not”) is 50%. The net number of correct responses (number correct – number incorrect) in 45 seconds is the dependent measure, with a floor of zero. This test is a measure of cognitive flexibility and reasoning.

**Progressive Matrices**

Matrix reasoning assessments (e.g., Raven [3]) require the participant to determine which stimulus most logically completes a multi-dimensional pattern. In this version of matrix reasoning, the participant is shown a 3x3 grid (each grid slot has width and height equal to 1/5 the window height) with abstract stimuli in the 8 upper-left slots. The task is to choose which of six possible answer choices best completes the pattern in the grid. The assessment is made up of 17 problems of increasing difficulty that are algorithmically generated from a set of parameters. The 17 problems are divided into three broad problem types: progression matrix, orbital/lateral movement, and Boolean logic. The first 12 trials involve progression matrix rules of increasing complexity. Characteristics of the stimuli that may change include shape, number, color, rotation angle, and size. These patterns may progress across the matrix horizontally, vertically, from upper-left to lower-right diagonal, or from lower-left to upper-right diagonal. Trials 13-15 involve orbital or lateral movement in which square grids or circular orbits are partially filled with elements that progress according to a lateral or rotational movement rule. Trials 16-17 involve Boolean logic in which spatial patterns are combined using Boolean operators such as AND, OR, and XOR. For each problem type, the correct answer is indicated regardless of whether the participant answers correctly. The assessment ends once the participant completes 17 trials or answers three consecutive trials incorrectly. The total number correct is the dependent measure. Matrix reasoning is considered a measure of problem solving and fluid reasoning (often referred to as fluid intelligence).

**Go/No-Go**

In this assessment, the participant must press the space bar in response to a target picture and withhold responding to distractor stimuli. The stimuli are chosen from a set of photos of fruit (apple, watermelon, pear, peach, orange, cantaloupe) and occupy roughly ¼ the height of the window. The identity of the target (e.g., a watermelon) is chosen randomly for a particular run of the assessment and shown to the participant prior to the beginning of the trials. On a given presentation, there is a 50% chance of the stimulus being a target. Each stimulus appears after a random delay varied between 1000 to 3000 msec to discourage anticipatory responding. The participant is told to respond as quickly as possible without making errors. The participant must respond to a “go” trial within 1500 msec. Timing and correctness feedback is given. The assessment ends when a participant responds to ten “go” trials. If a participant makes three errors (responding to “no-go” trials or failing to respond to “go” trials), the assessment is restarted. The dependent measure is the average reaction time on correct trials. This assessment is a measure of response inhibition and speed of processing.

**Arithmetic Reasoning**

This assessment [4] requires the participant to rapidly and accurately solve simple arithmetic problems that are written in words – for example, “Four plus two =”. The answer is input using the number keys on the keyboard (“6” in the current example). For addition and multiplication problems, operands are uniformly sampled from the integers in the range 1-9, and for subtraction and division problems, the second operand and solution are uniformly sampled from the integers in the range 1-9. The addition operator is used in the first five trials. Subsequent trials use addition 50% of the time. In non-addition trials, operators are chosen uniformly from subtraction, multiplication, and division. The assessment lasts 90 seconds, and the total number of correct responses in that time period is the dependent measure. It is considered a measure of problem solving ability.

**Two-Target Search**

In this assessment, the participant must identify two targets, while ignoring two distractors, which are spread across the field of view. In order to partially control for screen size and thus visual angle, prior to taking the assessment, the participant is instructed to hold a credit card-sized object up to the screen and adjust a slider until a representative rectangle matches in size. The display window is scaled accordingly. The participant is briefly presented with four circles containing letters. Each circle is 1 inch tall. Each of the four circles is distributed at a random angle along an invisible circle with a 2.5-inch radius centered in the middle of the screen. The minimum spacing between each stimulus circle is 0.75 inches in order to maximize the spread of visual attention [5]. Two of the letters are designated at the beginning of the session as target letters (e.g., “H” and “T”) selected from a set of six letters of similar shape (“F,” “H,” “K,” “T,” “X,” and “Z”). A fixation cross appears for 1000 msec, after which the four circled letters appear. After a brief presentation, the letters are obscured by visual masking noise made up of all six letters on top of each other (in order to prevent visual afterimage effects). Noise mask durations are equal to stimulus presentation durations. Next, the letters disappear but the circles remain. The participant is instructed to click on the two circles that had contained the target letters. Difficulty is manipulated using an up/down staircase method [6] with exponential scaling. The first trial begins with a 1000 msec presentation time and subsequent trials are adjusted by multiplying presentation time by 10^-0.15^ after a correct trial and 10^0.15^ after an incorrect trial and rounding up to the nearest 1/30 sec due to screen refresh rate quantization (possible range: 33.333-44666 msec). There are 12 trials total. The threshold presentation time derived from the staircase procedure is the dependent measure. The threshold presentation time is defined as the average of all reversal times (presentation times of trials in which the direction of the staircase switched direction due to answering correctly after a previously incorrect trial or vice-versa) and a potential final presentation time. The final presentation time is defined as the presentation time calculated for what would be the 13th trial, and is included in the average if the final trial was not a reversal trial (possible range: 15.849-63096 msec). This task is designed to be a measure of divided visual attention.

**References**

1. Milner B. Interhemispheric differences in the localization of psychological processes in man. Br Med Bull. 1971;27(3):272-7.

2. Baddeley AD. A 3 min reasoning test based on grammatical transformation. Psychon Sci. 1968;10(10):341-2.

3. Raven J. The Raven's progressive matrices: change and stability over culture and time. Cogn Psychol. 2000;41(1):1-48.

4. Deloche G, Seron X, Larroque C, Magnien C, Metz-Lutz MN, Noel MN, et al. Calculation and number processing: assessment battery; role of demographic factors. J Clin Exp Neuropsychol. 1994;16(2):195-208.

5. Sekuler R, Ball K. Visual localization: age and practice. J Opt Soc Am A. 1986;3(6):864-

6. Levitt H. Transformed up-down methods in psychoacoustics. J Acoust Soc Am. 1971;49(2B):467-77.
