# Supplementary material for: Enhancing Cognitive Abilities with Comprehensive Training: A Large, Online, Randomized, Active-Controlled Trial
Source: PLoS One. 2015 Sep 2;10(9):e0134467. doi: 10.1371/journal.pone.0134467 (PMC4557999; doi:10.1371/journal.pone.0134467)
Supplement: S1 File — The first supplementary analysis is an ANCOVA analysis that includes participants assigned to the control group who engaged in some cognitive training during the study period. The second supplementary analysis describes how engagement time is estimated in the two conditions and provides a paired-matching analysis that controls for the total time spent engaging with the two conditions. The third supplementary analysis includes an outlier removal procedure. (DOCX) [file pone.0134467.s006.docx]

**S1 Additional Analyses.** The first supplementary analysis is an ANCOVA analysis that includes participants assigned to the control group who engaged in some cognitive training during the study period. The second supplementary analysis describes how engagement time is estimated in the two conditions and provides a paired-matching analysis that controls for the total time spent engaging with the two conditions. The third supplementary analysis includes an outlier removal procedure.

**ANCOVA analysis including participants assigned to the control condition who engaged with cognitive training**

This analysis adds back control participants who were removed for having completed some amount of cognitive training during the study (n=330). For this analysis, their pre- and post-test scores were included with the rest of the control group data, and the ANCOVA models are otherwise identical to those used in the main text. Table A provides updated demographics for the control group. Table B provides new baseline and change score means for the control condition with these participants included along with the statistics for the main effect of treatment group from the new ANCOVA models, and is comparable to Table 2 in the main text. The pattern and magnitude of results from this analysis is quite similar to that observed in the previous analysis. For the primary outcome (change in Grand Index score), the group effect remained significant, with cognitive training participants improving more than control participants (t(5042) = 8.70, p < 10^-15^, d = 0.243, 95% confidence interval = [0.188, 0.298]). Thus, the results and conclusions of this study remain if participants who were excluded from the control condition are included in the analysis.

**Table A.** **Demographic information including the control participants who engaged with cognitive training during the study period.**

|  | **Crosswords Control** | **Cognitive Training** | **p-value** |
| --- | --- | --- | --- |
| **Age** |  |  |  |
| Mean (sd) | 38.6 (14.8) | 39.4 (15.2) | 0.17 |
| % 18-34 | 52.1% | 49.3% |  |
| % 35-54 | 29.9% | 30.6% |  |
| % 55 and older | 18.0% | 20.1% |  |
| **Gender**  % Female (% unreported) | 53.4% (2.1%) | 54.3% (2.5%) | 0.39 |
| **Education** |  |  | 0.25 |
| % high school graduate or less | 10.8% | 11.3% |  |
| % with some college | 24.6% | 23.2% |  |
| % with a bachelor’s degree | 32.7% | 30.2% |  |
| % with advanced degree (Masters, PhD, or Professional degree) | 27.5% | 28.9% |  |
| % unreported | 4.4% | 6.3% |  |

P-values are based on Kolmogorov-Smirnov test for age, and chi-square analyses for gender and education based on participants who reported this information.

**Table B.** **Results of ANCOVA analysis including the control participants who engaged with cognitive training during the study period.**

|  | Crossword Puzzles | | Cognitive Training | | Between Groups Difference in Change Means | |
| --- | --- | --- | --- | --- | --- | --- |
|  | Baseline  Mean (sd) | Change  Mean (sd) | Baseline  Mean (sd) | Change  Mean (sd) | p Value | Cohen’s *d* Effect Size  (95% Confidence Interval) |
| Forward Memory Span | 100.49 (13.77) | 0.24 (15.76) | 99.50 (14.16) | 2.73 (16.13) | p < 10^-6^ | 0.140  (0.085, 0.195) |
| Reverse Memory Span | 100.42 (13.91) | 0.78 (15.90) | 99.65 (13.99) | 2.57 (16.39) | p = 0.0007 | 0.096  (0.041, 0.152) |
| Grammatical Reasoning | 100.87 (14.58) | 2.76 (13.91) | 99.36 (14.78) | 2.27 (14.09) | p = 0.001 | -0.090  (-0.145, -0.035) |
| Progressive Matrices | 100.28 (14.52) | 1.29 (15.05) | 99.66 (14.90) | 3.02 (15.86) | p = 0.0002 | 0.104  (0.049, 0.159) |
| Go/No Go | 100.04 (14.85) | 1.93 (15.07) | 99.96 (15.10) | 4.00 (15.73) | p < 10^-7^ | 0.153  (0.098, 0.208) |
| Arithmetic Reasoning | 101.04 (14.86) | 0.66 (10.22) | 99.08 (14.87) | 3.64 (10.78) | p < 10^-15^ | 0.252  (0.197, 0.306) |
| Two-Target Search | 100.45 (14.89) | 0.30 (17.89) | 99.60 (15.03) | 1.28 (19.23) | p = 0.39 | 0.024  (-0.031, 0.079) |
| Grand Index | 100.95 (14.77) | 2.14 (10.64) | 99.15 (15.16) | 5.24 (12.00) | p < 10^-15^ | 0.243  (0.188, 0.298) |

Means and standard deviations of baseline and change scores are the unadjusted summary statistics. Significance levels and effect sizes are based on ANCOVA models controlling for pre-test means.

**Engagement time**

Here we describe a procedure for estimating how much time each participant spent actively engaging with either the cognitive training intervention or the crossword control condition.

**Cognitive Training**

As noted in the main text, the cognitive training program consisted of 49 tasks. Many tasks are timed to a specific length (e.g., 2 minutes), while a small number of others are untimed. As the training tasks are relatively short (between 45 seconds to 6 minutes) and require consistent engagement for good performance, using the total amount of time elapsed for each task can be considered a reasonable measure of the amount of time participants spent engaging with them.

To account for rare cases in which elapsed time was not properly recorded and cases in which participants left tasks open on their computer without completing them, we used the median recorded time elapsed for each task over all sessions in the dataset rather than the empirically recorded time elapsed for a specific session. These median values were then multiplied by the number of times each participant completed each task and summed for each participant to obtain an estimate of total time trained.

The majority of cognitive training participants (2,292) trained exclusively using the Lumosity web application. An additional 372 also completed at least one session of training on the mobile-phone-based Lumosity program, which included a subset of the tasks included in the web application. Times for mobile training tasks were estimated separately. The version of the mobile application available during the study period did not record time elapsed. However, subsequent versions of the mobile application did report these times. Thus, estimates of training time from the mobile training tasks were taken from usage data of the corresponding games from the updated app. One task (Memory Match) from the version of the mobile application used in this study was not included in the new application, so the time from the web version of this game was used. Since this game is timed at 45 seconds, this substitution should not result in a loss of precision in estimating training time.

**Crossword Control**

The total amount of time in the crosswords control condition was estimated based on the amount of time participants could be considered actively engaged with a puzzle while the crossword application was loaded. The crossword application reported a “progress” event every thirty seconds that the page was open, and a “submit” event if and when the participant submitted a puzzle as completed. These events included the number of cells of the crossword that the participant had filled along with the number of cells containing an error.

In order to differentiate periods of time in which a participant was likely searching for new words from those in which he or she likely left the application open and was not working on the puzzle, we selected a threshold over the number of progress windows that could pass without changes entered in the puzzle before the participant was considered idle. Beyond this threshold, we discounted any additional time elapsed until there was a change in either the number of cells filled or number of errors, indicating that the participant had resumed working on the puzzle. We chose six events (approximately three minutes) as our threshold based on the idea that this was a reasonable length of time for a participant to be actively searching for a word. Other thresholds between 2 and 10 windows (approximately 1 minute and 5 minutes respectively) were also tested, and in all cases, the median total time engaged with crosswords was greater than the 12.2 hours obtained for the cognitive training condition (range = [12.5, 14.7]) while the mean total time for crosswords was lower than the 16.1 hours obtained for the cognitive training condition (range = [12.0, 14.4]).

**Matched samples analysis**

To control for differences in the distribution of total engagement time between the two groups, an additional matched samples analysis was performed. In this analysis, results from participants with equivalent amounts of engagement time in the two groups were matched and compared.

As there were more fully evaluable participants in the cognitive training condition compared to the control condition, each control participant was matched to the treatment participant who most closely matched his or her estimated total engagement time (via the Matching package in R). Two different matching procedures were used. The first procedure matched with replacement, allowing different control participants to be matched with the same cognitive training participant multiple times if this provided the best match. Occasionally ties occurred, so 100 runs of this procedure were recorded, with ties broken randomly. The second procedure was identical to the first except that participants were matched without allowing replacement. As this procedure is sensitive to the order in which control participants are submitted to the procedure, it was repeated 1,000 times in order to obtain a range of estimates. After each run, the same ANCOVA models for the primary outcome measure and dose response effects described in the Results section of the main text were fit to the resulting dataset, providing a range of model coefficients and significance values.

The ANCOVA model for the primary outcome measure obtained a significant effect of group in all 100 datasets matched with replacement (*d* range = [0.203, 0.209], all p < 10^-10^) and all 1000 datasets matched without replacement (*d* range = [0.231, 0.241], all p < 10^-12^). The ANCOVA model for the dose-response effect based on active days also obtained a significant group-by-active-days interaction for all 100 datasets matched with replacement (B range = [0.052, 0.060], p = [0.0003, 0.002]) and for all 1000 datasets matched without replacement (B range = [0.043, 0.048], p = [0.011, 0.024]). These results indicate that differences in the distributions of total training time between the groups cannot account for the larger gains in aggregate cognitive performance for the cognitive training group compared to the crosswords control group.

**Outlier Removal Analysis**

As described in the Results, our primary analysis did not remove outlier values from the neurocognitive assessment scores. To examine any possible effects of outliers on the results, a secondary analysis was performed on the assessment change scores. For each assessment, the pooled standard deviation across pre- and post-tests raw scores (prior to normalization) was calculated, and scores that were outside the range of three standard deviations above or below the mean were removed. Table C provides the ranges of raw scores on each assessment before and after outlier removal. For the analysis of the primary outcome measure (change score on the Grand Index), participants with raw assessment scores outside the range of three standard deviations on one or more of the seven assessments were removed.

**Table C.** Ranges of raw assessment scores before and after removing outlying values.

|  | Range of raw scores before removing outlying values | Range of raw scores after removing outlying values |
| --- | --- | --- |
| Forward Memory Span  span length | 0 - 10 | 3 - 9 |
| Reverse Memory Span  span length | 0 - 10 | 3 - 8 |
| Grammatical Reasoning  net correct responses, floored at 0 | 0 - 30 | 0 - 22 |
| Progressive Matrices  number of correct responses | 0 – 17 | 1-17 |
| Go/No Go  mean RT on go trials | 238 - 1170 msec | 238 - 644 msec |
| Arithmetic Reasoning  number of correct responses | 0 - 35 | 1 - 29 |
| Two-Target Search  threshold presentation time | 15.9 – 16165.4 msec\ | 15.9 – 1123.4 msec |

Reverse Memory Span was the only assessment with significantly more participants removed from the treatment condition compared to the control condition (X^2^ = 5.10, p = .024, uncorrected, all other X^2^ < 1, p > .3), although the actual differences was small (1.6% from the control condition vs. 2.6% from the treatment condition) The procedure did not alter any of the main findings. Table D provides details on the number of participants removed from each condition via this procedure and revised ANCOVA results and between-group effect sizes.

**Table D.** **Participant removal rates and revised ANCOVA results from outlier removal analysis.**

|  |  | | Between-Group  Difference in Change Means | |
| --- | --- | --- | --- | --- |
|  | Control N remaining  N removed (percent) | Treatment N remaining  N removed (percent) | p Value | Cohen’s *d* Effect Size  (95% Confidence Interval) |
| Forward Memory Span | 2,011  37 (1.8%) | 2,621  46 (1.7%) | p < 10^-6^ | 0.149  (0.091, 0.207) |
| Reverse Memory Span | 2,015  33 (1.6%) | 2,597  70 (2.6%) | p = 0.0001 | 0.114  (0.056, 0.172) |
| Grammatical Reasoning | 2,039  9 (0.4%) | 2,659  8 (0.3%) | p = 0.005 | -0.082  (-0.14, -.024) |
| Progressive Matrices | 2,045  3 (0.1%) | 2,663  4 (0.1%) | p = 0.0001 | 0.113  (0.055, 0.171) |
| Go/No Go | 2,005  43 (2.1%) | 2,608  59 (2.2%) | p < 10^-6^ | 0.156  (0.098, 0.215) |
| Arithmetic Reasoning | 2,041  7 (0.3%) | 2,652  15 (0.6%) | p < 10^-15^ | 0.246  (0.188, 0.303) |
| Two-Target Search | 2,028  20 (0.1%) | 2,645  22 (0.8%) | p = 0.31 | 0.03  (-0.028, 0.088) |
| Grand Index | 1,919  129 (6.3%) | 2,480  187 (7.0%) | p < 10^-15^ | 0.267  (0.208, 0.326) |

Significance levels and effect sizes are based on ANCOVA models controlling for pre-test means.
