# Supplementary material for: Enhancing Cognitive Abilities with Comprehensive Training: A Large, Online, Randomized, Active-Controlled Trial
Source: PLoS One. 2015 Sep 2;10(9):e0134467. doi: 10.1371/journal.pone.0134467 (PMC4557999; doi:10.1371/journal.pone.0134467)
Supplement: S1 Protocol — (PDF) [file pone.0134467.s007.pdf]

PROTOCOL

A. Introduction and Background

Cognitive training is a promising approach to enhancing brain function, but the generalizability and impact of training outcomes are still controversial, especially in normal, healthy adults (Owen, et al., 2010). Most researchers agree that cognitive training can lead to gains in specific trained skills and abilities (e.g., Ball, et al., 2002). However, studies examining transfer of training benefits to skills and abilities not explicitly trained have yielded mixed results. While some studies have found that cognitive training can produce improvements in untrained tasks (Jaeggi, et al., 2009; Klingberg, 2010; Smith, et al., 2010; Edwards, et al., 2009; COGITO; Kesler, et al., 2011; Finn and MacDonald, 2011), others have failed to demonstrate transfer (Owen, et al., 2010). The specifics of the training, control task, assessments, protocol and sample involved in the study are likely all critical factors in determining which studies result in transfer and which do not. As different researchers have used widely different study designs, it is difficult to pinpoint the key determinants of transfer. In addition, most studies of cognitive training have been underpowered, utilizing small sample sizes that further cloud the interpretation of results.

Large, controlled, repeatable cognitive training study designs are needed to uncover the necessary conditions for successful transfer. Running large, controlled trials of cognitive training exercises in the traditional laboratory-based setting is costly, time consuming and tedious. In person neuropsychological testing requires trained professionals and can take many hours per study participant. Training in a laboratory is even more resource intensive, often involving on-site proctors and typically requiring frequent travel to the study site for participants. When participants are sourced only from the location surrounding the study site (most typically a university or hospital setting), the resulting convenience sample is naturally skewed toward the demographics of the local community, impairing the ability to generalize the findings of the study.

To address these limitations, we propose to conduct a series of very large, online, randomized, controlled trials of cognitive training. The study will use the Lumosity training program for the intervention group and a crossword puzzle sham training active control group. Assessments will be administered immediately before and immediately after a 10-week training period. The assessments include a battery of standard neuropsychological assessments that have been optimized for online administration and a questionnaire probing for changes in everyday cognitive failures.

B. Study Design and Procedures

The study is a two-arm, randomized, active-controlled intervention trial conducted online. No in-person contact will occur between study staff and participants. Participants will be volunteers who choose to click on a link provided in an invitation email sent to a subset of Lumosity users fitting the inclusion and exclusion criteria. From the link, participants will be provided with the consent form, and those who give their informed consent and agree to participate in the study

will then be randomly assigned to either the cognitive training (CT) intervention group or the crossword puzzle active control (AC) group. Those who choose not to participate will maintain their status as Lumosity free users.

Prior to beginning the training, participants will be given a questionnaire probing for changes in everyday cognitive failures, called the Real World Cognition Survey (RWCS), and a battery of standard neuropsychological assessments that have been optimized for online administration, called the Brain Performance Test (BPT). This initial assessment takes approximately 20 minutes to complete. See attached screenshots from the RWCS and BPT.

Following the pre-training assessment, participants will be instructed to begin their training. Participants will be instructed to login to the Lumosity website at least 5 days per week for 10 weeks. Upon logging in, participants will automatically be directed to the appropriate training -- CT or AC. Each daily training session will last approximately 15 minutes, for both CT and AC groups.

At the end of the 10-week training period, all participants will complete the RWCS and BPT. At this point participants who completed all parts of the study will be emailed a free 6-month activation code for Lumosity.com.

C. Inclusion/Exclusion Criteria

Inclusion criteria include being a registered Lumosity user, having access to a networked computer and being able to train at least 15 minutes per day for 5 days a week for 10 weeks. Exclusion criteria include being under 18 years of age.

D. Recruitment of Subjects

Potential volunteers will be invited to participate in the study by clicking on a link provided in an invitation email (see attached) sent to a subset of 35, 000 Lumosity users who meet the inclusion and exclusion criteria for the study. This subset of users will be selected at random. We anticipate that some percentage of users who receive the invitation email will enroll in the study, and a some percentage of those enrolled will complete the full training study. Our goal is to have 10, 000 participants complete the study. For reference, our user-base is currently 40 million subscribed users.

E. Consent Process and Timing

Participants will give their informed consent prior to beginning the training study. They will read the informed consent form online and indicate their consent by clicking a radio button (see attached). Participants will have unlimited time to read through the online form, and will have the opportunity to contact the researchers to ask questions before consenting to participate.

F. Risks, Discomforts, and Benefits to Subjects

There are no known risks as a result of participation in this study. There is the possibility that participants may experience some physical fatigue as result of result of cognitive training, but not any more so than from routine computer use. Participants will be able to take breaks and/or discontinue play as needed. Because participants will have already signed up as free Lumosity users, they will have already provided their contact information for our database. Therefore, there is little to no additional risk of a security breach by participating in the study. As with all of our user data, we will minimize this risk of a security breach by not using the participant's name to identify them on study records. All electronic data will be kept on a secure, encrypted server that is only accessible by Lumosity's research team.

There is a potential benefit of improved cognitive functioning from either or both the cognitive training intervention and the crossword active control intervention. Knowledge from this study will potentially advance research efforts surrounding online cognitive training and ultimately may lead to a better training product.

G. Money

Participants will not receive monetary compensation, nor will they incur any costs.

H. Data Analysis and Statistical Analysis

The data that is stored as part of the study includes demographic variables, assessment performance, and training data. Demographic variables are provided at will by Lumosity users as part of the standard user experience, and include date of birth, gender, and education level. Assessment performance variables include relevant individual raw variables that differ for each assessment, such as number correct, memory span, and presentation time threshold. They also include scaled equivalents of these raw variables based on an earlier reference population. Training data includes information about the timing and performance of each exercise. In the case of Lumosity training, the training variables include an identifier for the game, a timestamp, a game score, a scaled game score, and a variety of game-specific variables that differ depending on the game, such as response times, number of correct responses, and other variables.

All data is stored in a secure MySQL database and/or secured Amazon S3 buckets as part of the Lumosity production website and analytics backend. The Lumosity website has implemented and maintains reasonable security procedures and practices to protect against the unauthorized access, use, modification, destruction or disclosure of users' personal information. For example, we use one or more of SSL encryption, firewalls and self-updating anti-virus software.

The main dependent variables of interest in this study are the amount of improvement on the assessments. The main independent variables of interest are the training condition (crossword puzzles vs. Lumosity intervention) and the amount of training each participant completed. Additional variables including age, gender, level of education and initial performance on the assessments will also likely be included as covariates in order to control for their effects.

The goal of the analysis will be to discern whether the amount of the improvement on the assessments differs depending on the training condition, and whether this difference depends on the amount of training participants' completed. If Lumosity training is more effective than the crossword control condition at improving performance on the assessments, we would also predict that this difference would be dose-dependent. This hypothesis would predict an interaction of training condition and time spent training, wherein increased time spent training on Lumosity should predict larger improvements on the assessment battery than increased time spent training on crossword puzzles.
